# Effects of impacted mandibular third molar surgery performed with piezosurgery and conventional systems on postoperative sequelae and quality of life: a randomized controlled trial

**DOI:** 10.1186/s40902-026-00502-2

**Published:** 2026-01-30

**Authors:** Izzet Acikan, Aykut Can Balkanlioglu

**Affiliations:** Kahramanmaras Sutcu Imam University, Kahramanmaras, Turkey

**Keywords:** Impacted third molars, Mouth opening, Pain, Piezoelectric surgery, Quality of life, Swelling

## Abstract

**Background:**

Although piezosurgery has become a common technique in maxillofacial procedures—including third molar extractions—its advantages over conventional rotary instruments regarding postoperative recovery are still debated. Therefore, this randomized clinical trial aimed to evaluate and compare postoperative complications following the removal of impacted mandibular third molars using either piezosurgery or conventional rotary instruments.

**Methods:**

This randomized controlled clinical trial included a total of 50 participants who were randomly allocated into two groups: the piezosurgery group and the conventional surgery group. Postoperative outcomes were assessed in patients who underwent surgical extraction of impacted mandibular third molars using one of these two methods. Pain levels were recorded daily using a Visual Analog Scale (VAS), whereas swelling, mouth opening, and oral health–related quality of life were evaluated before surgery and again on postoperative days two and seven.

**Results:**

The piezosurgery technique resulted in a faster reduction of postoperative swelling on the second day; however, by the seventh day, no statistically significant differences were found between the two surgical techniques with respect to postoperative swelling. Similarly, there were no significant variations between the piezosurgery and conventional methods in terms of postoperative pain, mouth opening, or oral health–related quality of life throughout the observation period.

**Conclusion:**

Piezosurgery appears to offer a potential advantage; however, apart from reducing swelling, it does not demonstrate any significant superiority in terms of mouth opening, pain, or quality of life. Further standardized, large-scale randomized controlled studies are needed to obtain stronger evidence regarding the efficacy of both techniques.

## Background

Surgical removal of impacted third molars represents one of the most frequently performed operations in oral and maxillofacial practice [1]. Impacted third molars may cause complications such as pericoronitis, pain, infection, cysts, and damage to adjacent teeth, making their extraction necessary [2]. Postoperative issues like pain, swelling, trismus, infection, and nerve injury can occur, affecting patients’ quality of life, with reported incidence rates ranging from 0% to 30% [3–5].

Minimally invasive techniques help reduce such complications [6]. Various methods—including new flap designs, drainage, prophylactic antibiotics, coronectomy, corticosteroids, and alternative osteotomy devices like piezosurgery—have been explored to minimize postoperative morbidity [6–8]. Traditionally, bone removal in third molar surgery has been performed using rotary burs, which are efficient but may generate excessive heat and cause trauma to nearby tissues, leading to complications such as pain, bleeding, and delayed healing [1, 2, 6, 9–11].

To overcome these drawbacks, piezosurgery was developed as a precise and safe alternative for bone cutting [7, 12, 13]. Based on ultrasonic microvibrations, piezosurgery enables selective cutting of mineralized tissue while preserving soft tissue and blood supply [7, 12, 14]. Since its introduction in maxillofacial surgery, it has been proposed to reduce postoperative morbidity. However, studies comparing piezosurgery with conventional rotary systems have yielded conflicting results—some report reduced pain, swelling, and trismus, while others show no significant difference [2, 3, 6, 8, 11, 13, 15–18].

Importantly, the heterogeneity observed in the existing literature may be attributed to methodological variations, inconsistent postoperative assessment time points, and the predominant use of two-dimensional or linear methods for evaluating facial swelling. Moreover, relatively few randomized controlled trials have simultaneously assessed postoperative pain, mouth opening, and oral health–related quality of life using validated instruments, while objectively quantifying postoperative swelling through three-dimensional volumetric analysis.

Therefore, the present randomized controlled clinical trial was designed to compare the effects of piezosurgery and conventional rotary techniques on postoperative pain, facial swelling, mouth opening, and oral health–related quality of life following the surgical removal of impacted mandibular third molars, using standardized assessment time points and objective three-dimensional volumetric measurements.

The null hypothesis (H0) of this study was that there would be no statistically significant differences between piezosurgery and conventional rotary techniques with respect to postoperative pain (VAS), facial swelling (volumetric analysis), mouth opening, and OHIP-14 scores following impacted mandibular third molar surgery.

## Materials and methods

### Study design and ethical approval

This two-arm prospective randomized controlled trial was designed to assess and compare postoperative parameters—such as swelling, pain, mouth opening, and oral health–related quality of life—following the extraction of impacted mandibular third molars. The study protocol was approved by the Ethics Committee of Harran University (approval code: HRÜ/*25*.*07*.*46*). All procedures were carried out in accordance with the ethical standards of the Declaration of Helsinki, given that the research involved human participants. Prior to participation, each subject provided written informed consent. The trial was registered on ClinicalTrials.gov with the registration number NCT07185620.

The research was carried out at the Department of Oral and Maxillofacial Surgery, Faculty of Dentistry, Kahramanmaras Sutcu Imam University. Fifty patients were enrolled in the study, and the impacted teeth operated on were mandibular third molars with varying degrees of surgical difficulty. Tooth angulation, depth of impaction, and the relationship to the mandibular ramus were evaluated preoperatively using panoramic radiographs and classified according to Winter’s and Pell and Gregory’s classifications. Surgical difficulty was assessed using the Pederson difficulty index, based on which cases were classified as minimally difficult, moderately difficult, or very difficult. To minimize bias related to surgical complexity, patients with comparable difficulty levels were matched in pairs and subsequently randomized to either the piezosurgery or conventional surgery group using a sealed-envelope randomization technique, in which two envelopes representing the two surgical groups were prepared by the surgeon’s assistant and one patient from each pair selected one envelope. The study was conducted in a single-blind design. The participants were blinded to group allocation, while the surgeon and assistant were aware of the interventions. The outcome assessor (who evaluated pain, swelling, and mouth opening) was blinded to the treatment groups to minimize assessment bias.

Inclusion criteria included being over 18 years of age, having an indication for surgical removal of an impacted mandibular third molar, absence of active infection such as acute pericoronitis, and willingness to participate in the study.

Exclusion criteria included smoking more than 10 cigarettes per day, diabetes, immunodeficiency, bleeding diathesis, systemic conditions such as dyspepsia or peptic ulcer, and any pathology that could affect wound healing. These criteria were established to ensure a homogeneous and healthy adult sample.

### Power analysis

The sample size was determined based on the expected effect size (Cohen’s d) derived from clinically meaningful differences in postoperative pain scores. Previous studies have reported a standard deviation of approximately 2.0 points (VAS, *0*–*10*), and a minimum clinically important difference of about 1.6 points was considered relevant, corresponding to a large effect size (Cohen’s d ≈ *0.8*). According to a sensitivity analysis conducted using GPower (two-tailed independent samples t-test, α = *0.05*, power = *0.80*), a total of 50 participants (*25* per group) would allow detection of effects of at least d ≈ 0.79. All calculations were performed using GPower software (version *3.1*, Düsseldorf, Germany).

### Surgical procedure

All surgical procedures were performed by a single operator under local anesthesia. An inferior alveolar nerve block anesthesia was achieved using 2% lidocaine with 1:100,000 epinephrine, followed by buccal and lingual infiltrations to ensure adequate anesthesia. Surgical access was obtained using a modified envelope flap.

In the study group (piezosurgery group), osteotomy was carried out using a piezoelectric surgical system specifically designed for bone cutting. The procedure was performed with dedicated bone-cutting tips operating at ultrasonic microvibration frequencies, which allow selective cutting of mineralized tissue while minimizing trauma to adjacent soft tissues. Continuous sterile saline irrigation was applied throughout the osteotomy process to prevent thermal damage and to ensure adequate cooling of the surgical site.

In the control group (conventional surgery group), osteotomy was performed using a surgical handpiece operating at a rotational speed of 20,000–40,000 rpm with a #10 round bur. This technique enabled efficient bone removal through rotary instrumentation. Continuous sterile saline irrigation was applied throughout the procedure to limit heat generation, reduce the risk of thermal injury to the surrounding bone and soft tissues, and ensure adequate cooling of the surgical site. Apart from the osteotomy method, all surgical steps were standardized between the two groups.

Tooth luxation and extraction were performed using conventional elevators and forceps. When required for tooth removal, odontotomy (crown and/or root sectioning) was carried out following the same surgical principles in both groups; piezoelectric tips were used in the piezosurgery group, whereas rotary burs were used in the conventional group. After tooth extraction, bone margins were smoothed, and the surgical site was thoroughly irrigated with sterile saline solution.

Hemostasis was achieved in all cases, and the mucoperiosteal flap was repositioned and closed primarily using standard suturing techniques. All patients received a standardized postoperative medication protocol, including analgesics, antibiotics, and antiseptic mouth rinses. Postoperative instructions were provided uniformly to all participants.

### Imaging, alignment, and volumetric swelling analysis

To objectively evaluate postoperative facial swelling, a three-dimensional (3D) facial scanning method was used. Each patient underwent three scans: immediately before surgery (T*0*), on postoperative day two (T*1*), and on postoperative day seven (T*2*). All scans were performed in the same room, under identical lighting conditions, with the patient in a fixed position.

**Fig. 1:**
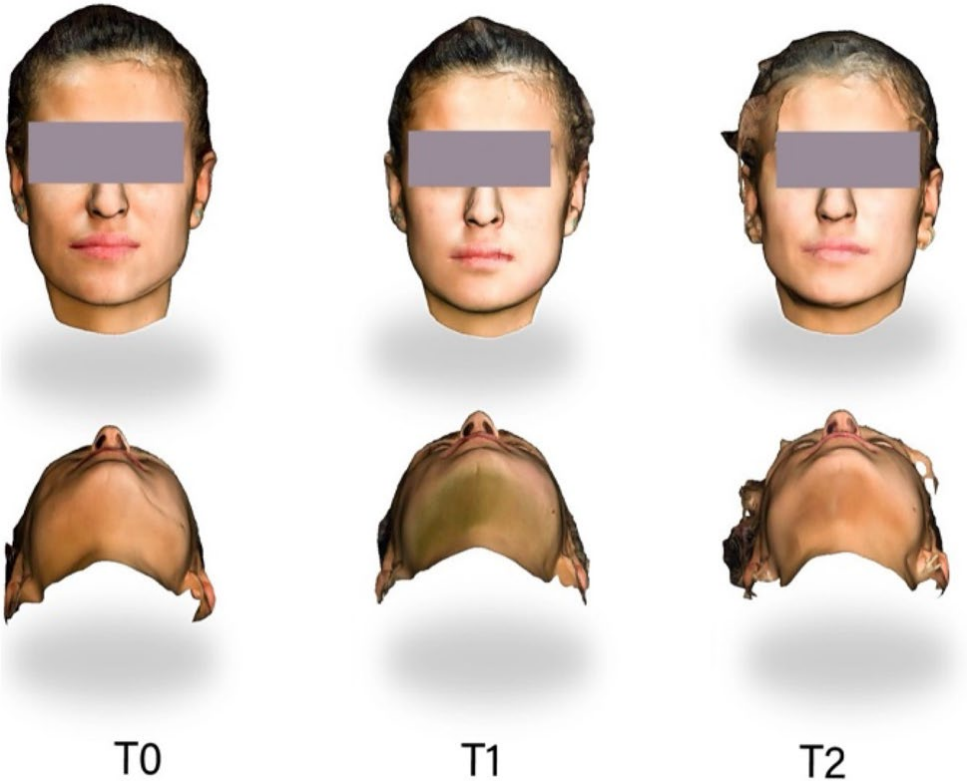
Three-dimensional facial scans obtained before and after mandibular third molar surgery. (T*0*) Baseline preoperative assessment; (T*1*) postoperative day 2; (T*2*) postoperative day 7. The upper row shows frontal views, while the lower row shows submento-vertical (worm’s-eye) views. Images were acquired using the Bellus3D Face App (STL format) and analyzed with 3D Slicer software

3D facial images were captured using the Qlone application (EyeCue Vision Technologies LTD.) running on an iPhone 14 with TrueDepth camera technology. This smartphone-based application provides a guided scanning process that enables quick and detailed 3D facial scans. At the end of each session, an stereolithography (STL) file representing the patient’s facial structure was generated (Fig. 1).

**Fig. 2:**
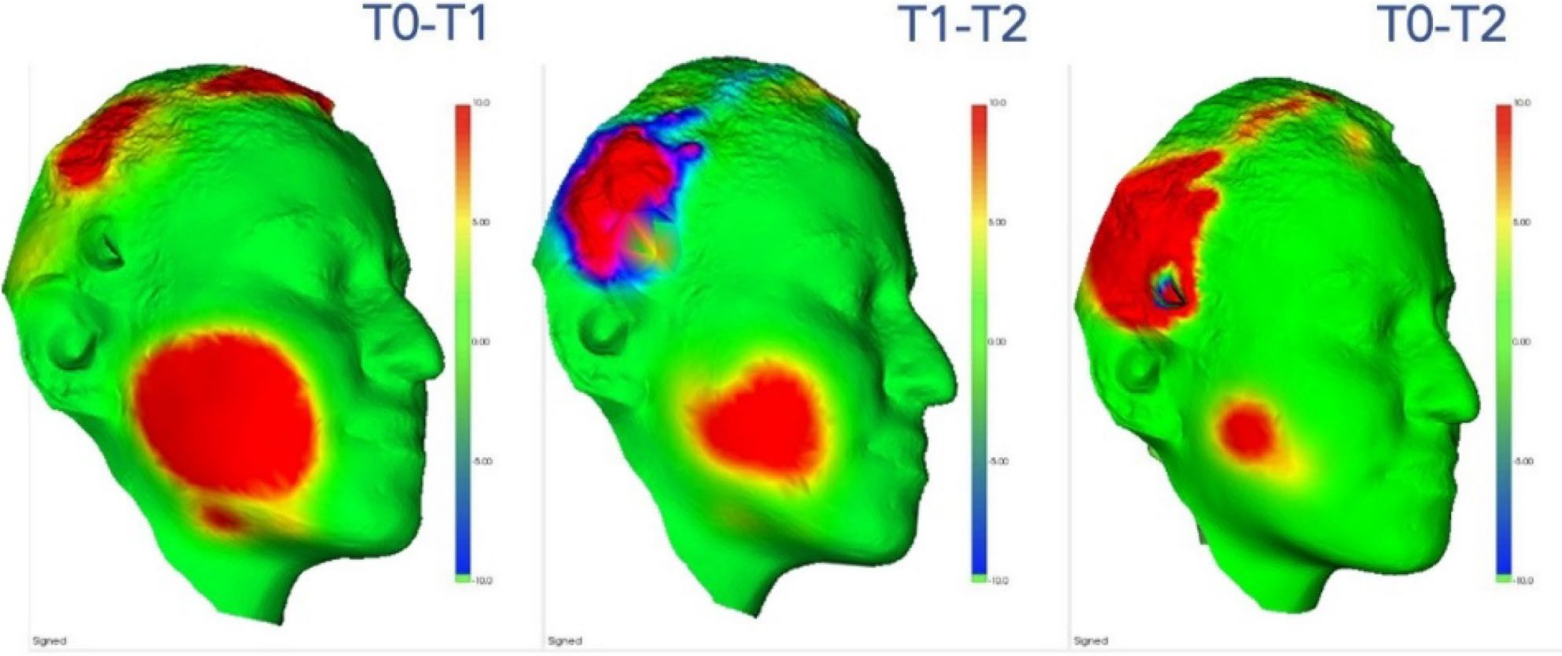
Color maps of facial volumetric changes at different time points following mandibular third molar surgery. (T*0*–T*1*) Preoperative vs. postoperative day 2; (T*1*–T*2*) postoperative day 2 vs. day 7; (T*0*–T*2*) preoperative vs. postoperative day 7. The color scale represents surface changes in millimeters (red: edema/volume increase, blue: volume decrease, green: minimal change). A progressive reduction of the red areas, representing postoperative edema, is clearly observed over time

The STL files were imported into 3D Slicer software for processing. T0 and T1/T2 scans were superimposed (aligned) within the software to detect surface changes. Alignment was performed using surface registration based on fixed anatomical facial landmarks. Only the surgical side of the face was analyzed, while movable regions such as hair and eyelashes were excluded (Fig. 2).

**Fig. 3:**
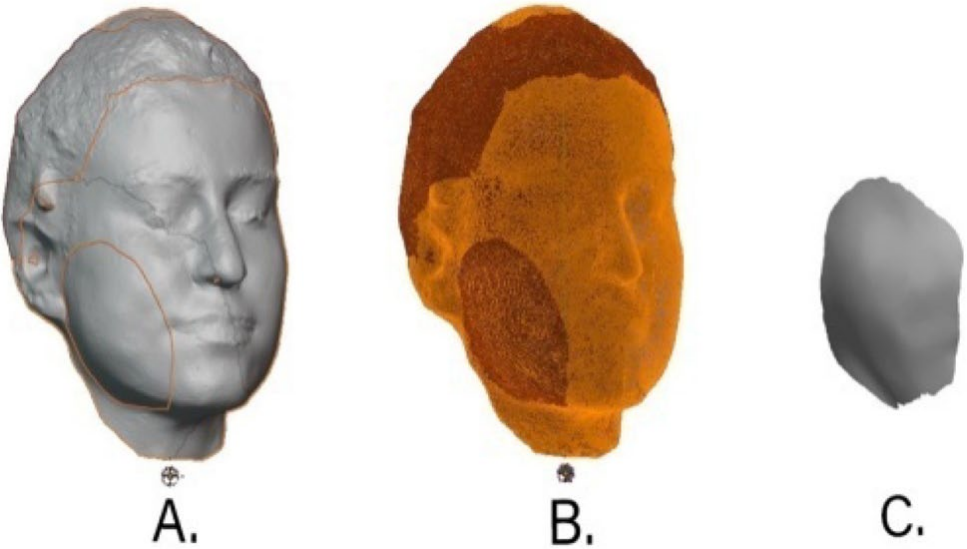
Workflow for volumetric isolation of postoperative swelling using Blender software (version 4.0). (**A**) Superimposition of preoperative (T*0*) and postoperative day 2 (T*1*) three-dimensional facial scans. (**B**) The volumetric difference between T0 and T1 models was extracted using the boolean cut function in Blender. During this process, the additional tissue volume representing postoperative edema was separated as an independent layer from the facial anatomy. This allowed the isolated swelling region to be clearly visualized and distinguished for subsequent quantitative evaluation. (**C**) The final isolated swelling volume was exported in stereolithography (STL) format for volumetric comparison and quantitative analysis

Aligned 3D facial models were then transferred to Blender 4.0 software (Blender Foundation, Amsterdam, Netherlands) to calculate volumetric differences. Using the Boolean difference method, the postoperative model was subtracted from the preoperative one, generating a 3D swelling object representing the excess volume. The geometry of the swelling object was checked and converted into a closed solid volume (Fig. 3).

### Swelling volume calculation

To determine the volume of irregularly shaped solid objects, CITUBOX software was used. This method provides high accuracy in volume measurements of irregularly shaped biological or inorganic samples where standard geometric volume calculations are not applicable.

The measurement procedure was performed as follows:

1. The sample was placed into the measurement module integrated with the CITUBOX software.
2. The software automatically calculated the required amount of resin for the measurement and recorded the relevant parameters in the interface.
3. The volume of resin used corresponded to the actual volume occupied by the irregular solid object — in other words, the resin’s fill capacity was directly proportional to the object’s volume.
4. The obtained resin volume (in mL) was accepted as the object’s volumetric value.

This technique provides repeatable and reliable volumetric measurements, particularly for irregular structures, and is more practical and less operator-dependent than traditional fluid displacement methods. Thus, the volumetric facial swelling for each patient was quantitatively determined as the difference between T0–T1 and T0–T2.

### Mouth opening

Preoperative and postoperative interincisal mouth opening was measured using a standard monoblock caliper. Participants were seated upright with the orbitomeatal plane parallel to the floor during measurement. The maximum unassisted midline interincisal distance was recorded in millimeters (mm). Measurements were taken at three time points: preoperatively (T*0*), on postoperative day two (T*2*), and on postoperative day seven (T*7*).

### Pain assessment

Pain intensity was assessed using a 10-cm Visual Analog Scale (VAS), where 0 indicated no pain and 10 represented the maximum pain imaginable. The VAS is a widely accepted and validated method for pain assessment in clinical studies [19]. Baseline VAS was recorded 20 min before surgery (T*0*), and the highest daily pain score was self-reported by patients on postoperative days one (T*1*) through seven (T*7*).

### Quality of life (Oral Health Impact Profile (OHIP)-14)

Participants’ oral health–related quality of life was evaluated using the Oral Health Impact Profile-14 (OHIP-14) questionnaire. OHIP-14 is the short-form version of the Oral Health Impact Profile originally developed by Slade and Spencer, designed to assess the social impact of oral disorders on quality of life [20]. The survey was administered before surgery (T*0*) and again on postoperative days two (T*2*) and seven (T*7*). Each question was rated on a four-point Likert scale, where 1 represented “never” and 4 represented “very often.” The cumulative score could range from 14 to 56, with higher values reflecting a greater negative impact on quality of life.

### Statistical analysis

The Shapiro–Wilk test was applied to examine whether the data followed a normal distribution. Since the data satisfied the assumption of normality, comparisons between the two groups at different time points were performed using the independent Student’s t-test. Temporal changes in post-operative pain measurements were evaluated by comparing group means across the respective time intervals. All analyses were conducted using Jamovi statistical software (version *2*.*3*.*28*; Sydney, Australia), and a significance level of *p* < 0.05 was adopted.

## Results

Fifty patients presenting to the Department of Oral and Maxillofacial Surgery, Faculty of Dentistry, Kahramanmaras Sutcu Imam University, with complaints related to impacted mandibular third molars were enrolled in this study. The study was conducted on 50 patients aged between 18 and 44 years (mean age *23.96* ± *6.86* in the piezosurgery group and mean age 23.88 ± 6.40 in the conventional group), consisting of 18 males (*36*%) and 32 females (*64*%) with mandibular impacted third molars.

In the evaluation of postoperative pain, no statistically significant differences were found between the piezosurgery and conventional groups in VAS scores at any time point (*p* > *0.05*). Despite the lack of statistical significance, the piezosurgery group demonstrated slightly lower VAS scores on days one and seven, whereas both groups exhibited identical scores on day six (*p* > *0.05*). On the remaining days, the conventional group showed marginally lower pain levels, though these differences were not statistically significant (*p* > *0.05*) (Table 1).

**Table 1:** Comparisons of visual analogue scale (VAS) scores of conventional surgery and piezosurgery

VAS	Conventionel Mean ± SD	Piezosurgery Mean ± SD	P*
N	25	25	
T0	2.96 ± 2.731	3.960 ± 3.494	0.265
T1	7.20 ± 2.483	6.520 ± 2.648	0.354
T2	5.80 ± 2.958	6.080 ± 2.798	0.732
T3	4.72 ± 2.525	5.000 ± 2.449	0.692
T4	3.44 ± 2.347	3.840 ± 2.249	0.541
T5	3.08 ± 1.977	3.600 ± 2.198	0.384
T6	2.12 ± 2.186	2.120 ± 1.691	1.000
T7	1.56 ± 1.828	1.200 ± 1.658	0.469

*Independent Student’s T-Test

In the assessment of mouth opening, no statistically significant differences were detected between the piezosurgery and conventional groups at the preoperative, second-day, or seventh-day evaluations (p > *0.05*). Nevertheless, the piezosurgery group exhibited slightly greater mouth opening values on postoperative days two and seven, although the differences did not reach statistical significance (*p* > *0.05*) (Table 2).

**Table 2:** Comparisons of mouth opening, swelling and OHIP-14 of conventional surgery and piezosurgery

*n*	Conventionel Mean ± SD	Piezosurgery Mean ± SD	P*
	25	25	
Mouth opening(T0)	5.300 ± 0.661	5.400 ± 0.491	0.547
Mouth opening(T2)	3.792 ± 1.123	3.912 ± 1.181	0.714
Mouth opening(T7)	4.708 ± 0.975	4.736 ± 1.102	0.925
Swelling(T0-T1)	35.329 ± 13.736	23.639 ± 7.513	< 0.001
Swelling(T0-T2)	1.094 ± 0.370	1.022 ± 0.389	0.506
OHIP-14(T0)	14.320 ± 11.194	16.960 ± 12.005	0.425
OHIP-14(T2)	21.000 ± 13.964	21.680 ± 13.331	0.861
OHIP-14(T7)	17.840 ± 10.877	18.520 ± 13.675	0.847

*Independent Student’s T-Test

In the evaluation of postoperative swelling, the piezosurgery group demonstrated a significantly lower degree of edema compared with the conventional group on the second postoperative day (*p* < *0.05*). By the seventh day, however, the difference between the two groups was no longer statistically significant (*p* > *0.05*) (Table 2).

Analysis of postoperative quality of life revealed no statistically significant differences between the piezosurgery and conventional groups on either the second or seventh postoperative day (*p* > *0.05*). However, although not statistically significant, the conventional group showed lower quality of life scores on both days. This suggests that, while not statistically significant, the conventional technique may have numerically improved the quality of life (Table 2).

## Discussion

This randomized clinical trial aimed to evaluate and compare postoperative outcomes following the extraction of impacted mandibular third molars using piezosurgery and conventional rotary techniques. Although this topic has been extensively discussed in the literature, Cicciù et al., in their meta-analysis, emphasized the high heterogeneity among published studies and showed through trial sequential analyses that the required information threshold for the Z-curve had not been reached. This indicates that stronger studies are still needed to obtain definitive conclusions [3, 21].

Postoperative complications such as pain, swelling, and trismus can vary according to multiple factors, including the patient’s general systemic health. To control for these variables, the present study included only systemically healthy adult participants, and all extractions were carried out by a single experienced surgeon. This approach was intended to reduce procedural variability and ensure that any differences observed could be attributed to the surgical method rather than to external influences [2]. In split-mouth studies, it is often difficult to find impacted teeth with the same level of difficulty even within the same patient [8]. Therefore, in this study, randomization was performed among different patients with teeth of similar difficulty levels.

Third molar surgery significantly affects patients’ quality of life, and every improvement in surgical technique contributes to patient satisfaction [8]. In the present study, the OHIP-14 scale was used to evaluate quality of life in both groups. Contrary to some previous studies, no significant difference was observed between the piezosurgery and conventional groups. Similarly, Menziletoğlu et al. used a general quality-of-life scale and reported findings consistent with ours, showing no statistically significant difference [22]. However, Demirci et al. used the OHIP-14 scale and found significantly better scores in the piezosurgery group [8]. Likewise, Piersanti and Goyal, using postoperative symptom severity scales, reported significantly better quality-of-life scores for piezosurgery [23, 24].

In the early postoperative phase, pain primarily arises from the body’s stress response and the inflammatory processes triggered by surgical trauma. In contrast, conditions such as osteitis, soft tissue inflammation, and lymph node involvement are typically responsible for pain that develops during the later stages of recovery [25]. Patients feel the most intense pain around the 5th hour on the day of surgery [6, 11]. Similar to our study, VAS values indicated very severe pain during the first 24 h. In our study, pain intensity decreased gradually over time. In several studies comparing conventional and piezosurgery techniques, pain was reported to be significantly lower in the piezosurgery group [7, 24, 26, 27]. In Mantovani’s study—considered the largest split-mouth investigation to date with 100 patients and surgeons of varying experience—although piezosurgery required more time for osteotomy, there was no difference in VAS pain scores on postoperative day two; however, In a previous study, pain intensity was reported to be significantly lower in the piezosurgery group on postoperative days 4 and 6 [28]. Erdem et al. observed that VAS-based pain scores were markedly higher in the conventional group from the sixth postoperative hour up to day 4, after which pain levels became comparable between the two groups [2]. In the present investigation, although patients in the conventional group experienced greater pain on days 1 and 7, the piezosurgery group showed slightly higher scores on the remaining days; however, these differences were not statistically significant. Similarly, other studies have also reported no significant variation in postoperative pain between conventional and piezosurgery approaches [23, 29–32]. These findings suggest that the subjective nature of pain perception, along with factors such as anxiety and individual pain thresholds, may account for the inconsistency in reported outcomes. Therefore, pain assessment tends to be less objective and more variable than measurable parameters like swelling and trismus [2, 8].

Postoperative swelling represents the inflammatory phase of wound healing; therefore, faster resolution of edema may indicate faster recovery. Many researchers have reported that osteotomies performed with piezosurgery heal faster than conventional techniques due to minimized trauma [6]. Goyal et al. found that piezosurgery significantly reduced postoperative swelling [24]. Jiang et al., in their meta-analysis, also reported a reduction in facial swelling with piezoelectric surgery, which was confirmed in our study [27]. Al Moraissi et al. reported significantly lower rates of postoperative complications with piezosurgery, attributing this to less bone damage and better hemostasis, leading to reduced edema risk [32]. Piersanti et al. and Sortino et al. also reported lower postoperative swelling in the piezosurgery group, although Sortino et al. found shorter operation times with rotary instruments [23, 33]. In our study, swelling was significantly lower in the piezosurgery group on postoperative day two; however, by day seven, although the swelling remained less in the piezo group, the difference was not statistically significant. However, some studies have reported that piezosurgery showed no superiority over conventional techniques at any measurement time in terms of postoperative swelling [7, 22, 29].

The use of rotary instruments for tooth sectioning combined with piezosurgery for bone removal may shorten the surgical time while preserving bone integrity and maintaining the benefits of piezosurgery. Although piezosurgery is less traumatic, it often requires prolonged soft tissue retraction, which may increase swelling and trismus. Several studies have linked longer operation times directly with increased postoperative morbidity after third molar surgery [25].

The main factor in the development of trismus is trauma during surgery. Studies have shown that trismus peaks on postoperative day two and generally resolves by the end of the first week [6]. Consistent with the literature, we measured mouth opening preoperatively and on postoperative days two and seven. In our study, although the piezosurgery group exhibited greater mouth opening on days two and seven, the difference between the groups was not statistically significant. Similarly, some studies have reported that piezosurgery has no superiority over conventional methods in terms of postoperative mouth opening. In addition, Sivolella et al. noted that surgical time was longer in procedures performed with piezosurgery [22, 23, 29, 30]. Conversely, Saraiva Amaral et al. reported better trismus outcomes in the piezo group, except for day three, when mouth opening restriction was greater [31]. Erdem et al. found significantly greater mouth opening in the piezo group on postoperative days one, three, and seven [2]. Mistry et al. reported a statistically significant increase in maximum interincisal distance in the piezo group on days one, three, five, and seven [34].

Current literature provides no strong evidence that either piezosurgery or rotary systems are superior in terms of postoperative pain, swelling, or mouth opening following third molar extractions [16]. The conventional method remains widely used due to operator familiarity, faster bone removal, and cost-effectiveness. However, piezosurgery procedures typically take longer due to slower bone cutting, which may limit its practicality in time-sensitive cases or among surgeons accustomed to rotary tools [1]. The prolonged operation time in the piezosurgery group may have offset some of its potential benefits. Although piezosurgery allows for a more atraumatic procedure, it requires longer soft tissue retraction, potentially contributing to greater pain, swelling, and trismus [22]. Another drawback of piezosurgery is its steep learning curve; excessive pressure on the tip can reduce cutting efficiency, convert energy into heat, and damage both bone and surrounding soft tissues despite its selective cutting ability [3].

The findings of this study, together with previous research, can be explained by the hypothesis proposed by Rullo et al. In that study, piezosurgery was reported to provide better postoperative outcomes in simpler extraction cases, but significantly worse postoperative outcomes in more complex cases [35]. The limitations of the present study include the inability to achieve double blinding and complete operator masking, the absence of documented surgical duration, and the use of a parallel-group rather than a split-mouth study design.

## Conclusion

Within the limitations of this study, the findings suggest that piezosurgery may be associated with reduced postoperative swelling. However, definitive conclusions regarding the superiority of one technique over the other cannot be drawn. Future studies should include more homogeneous patient groups in which simple and complex extractions are evaluated separately and should ideally be conducted by surgeons with comparable experience in both techniques. Although surgical duration was not recorded in the present study, the longer operative time commonly reported for piezosurgery may potentially offset some of its advantages. Therefore, further research should focus on optimizing piezosurgical protocols to reduce operation time without compromising clinical outcomes, which may improve patient-reported quality of life and increase the clinical applicability of piezosurgery in oral and maxillofacial surgery.

## Data Availability

The datasets generated and analysed during the current study are availbale from the corresponding author upon reasonable request.

## Abbreviations

VAS: Visual analog scale
3D: Three-dimensional
STL: Stereolithography
OHIP: Oral health impact profile
